# Beneficial effects of the combination of BCc1 and Hep-S nanochelating-based medicines on IL-6 in hospitalized moderate COVID-19 adult patients: a randomized, double-blind, placebo-controlled clinical trial

**DOI:** 10.1186/s13063-023-07624-2

**Published:** 2023-11-11

**Authors:** Maryam Hafizi, Somayeh Kalanaky, Saideh Fakharzadeh, Pegah Karimi, Atefeh Fakharian, Somayeh Lookzadeh, Esmaeil Mortaz, Maryam Sadat Mirenayat, Jalal Heshmatnia, Mehrdad Bakhshayesh Karam, Homa Zamani, Alireza Nadji, Mihan Pourabdollah Toutkaboni, Saeed Oraee-Yazdani, Mohammad Esmaeil Akbari, Hamidreza Jamaati, Mohammad Hassan Nazaran

**Affiliations:** 1 Department of Research and Development, Sodour Ahrar Shargh Company, Tehran, Iran; 2 Clinical Tuberculosis and Epidemiology Research Center, National Research Institute of Tuberculosis and Lung Diseases (NRITLD), Shahid Beheshti University of Medical Sciences, Tehran, Iran; 3 Department of Immunology, School of Medicine, Shahid Beheshti University of Medical Sciences, Tehran, Iran; 4 Chronic Respiratory Diseases Research Center (CRDRC), National Research Institute of Tuberculosis and Lung Diseases (NRITLD), Shahid Beheshti University of Medical Sciences, Tehran, Iran; 5 Functional Neurosurgery Research Center, Comprehensive Neurosurgical Center of Excellence, Shohada Tajrish, Shahid Beheshti University of Medical Sciences, Tehran, Iran; 6 Cancer Research Center, Shahid Beheshti University of Medical Sciences, Tehran, Iran

**Keywords:** COVID-19, BCc1, Hep-S, Nanochelating technology, IL-6, Cytokine storm syndrome

## Abstract

**Background:**

In the severe forms of COVID-19 and many other infectious diseases, the patients develop a cytokine storm syndrome (CSS) where pro-inflammatory cytokines such as IL-6 and TNF-α play a key role in the development of this serious process. Selenium and iron are two important trace minerals, and their metabolism is tightly connected to immune system function. Numerous studies highlight the role of selenium and iron metabolism changes in the procedure of COVID-19 inflammation. The immunomodulator effect of nanomedicines that are synthesized based on nanochelating technology has been proved in previous studies. In the present study, the effects of the combination of BCc1(with iron-chelating property) and Hep-S (containing selenium) nanomedicines on mentioned cytokines levels in hospitalized moderate COVID-19 patients were evaluated.

**Methods:**

Laboratory-confirmed moderate COVID-19 patients were enrolled to participate in a randomized, double-blind, placebo-controlled study in two separate groups: combination of BCc1 and Hep-S (*N* = 62) (treatment) or placebo (*N* = 60) (placebo). The blood samples were taken before medications on day zero, at discharge, and 28 days after consumption to measure hematological and biochemical parameters and cytokine levels. The clinical symptoms of all the patients were recorded according to an assessment questionnaire before the start of the treatment and on days 3 and discharge day.

**Results:**

The results revealed that consumption of the nanomedicines led to a significant decrease in the mean level of IL-6 cytokine, and at the end of the study, there was a 77% downward trend in IL-6 in the nanomedicine group, while an 18% increase in the placebo group (*p* < 0.05). In addition, the patients in the nanomedicines group had lower TNF-α levels; accordingly, there was a 21% decrease in TNF-α level in the treatment group, while a 31% increase in this cytokine level in the placebo was observed (*p* > 0.05). On the other hand, in nanomedicines treated groups, clinical scores of coughing, fatigue, and need for oxygen therapy improved.

**Conclusions:**

In conclusion, the combination of BCc1 and Hep-S inhibits IL-6 as a highly important and well-known cytokine in COVID-19 pathophysiology and presents a promising view for immunomodulation that can manage CSS.

**Trial registration:**

Iranian Registry of Clinical Trials RCT20170731035423N2. Registered on June 12, 2020.

**Supplementary Information:**

The online version contains supplementary material available at 10.1186/s13063-023-07624-2.

## Background

COVID-19 first appeared in China in early 2020 and quickly spread all around the world. This eventually made the World Health Organization (WHO) formally declare the disease as a “Global Pandemic” in March 2020. The virus that causes COVID-19 disease is called severe acute respiratory syndrome virus No. 2 (SARS-CoV-2) [1] which belongs to the coronavirus family [2, 3]. As soon as the virus enters the alveolar epithelial cells, it begins to multiply, triggering a chain of inflammatory and immune responses that lead to cytokine storm syndrome (CSS), lung tissue damage, and eventually acute respiratory distress syndrome [4]. Numerous studies have shown that interleukin-6 (IL-6) is the primary mediator of this process as a proinflammatory cytokine [5–7]. However, the concept of CCS and the critical role of IL-6 is not limited to complications of COVID-19 but has been observed in diverse infections and immune-mediated diseases [8].

The available therapeutic interventions for COVID-19 can be classified as antiviral drugs, anti-inflammatory drugs, monoclonal antibodies, and plasma therapy, the efficacy of which is being studied in various clinical studies [9], and according to recent WHO reports, there is no certain proof of the therapeutic effects of these medicines (https://www.who.int/publications/i/item/WHO-2019-nCoV-therapeutics-2021.1.) other than antiviral therapies, which can improve clinical outcomes in COVID-19 patients when administered early after diagnosis. One of the major challenges of many of these medicines is their side effects [10], which are sometimes exacerbated in combination therapies [11].

Microelements are vital elements whose metabolism modifications substantially affect the immune system responses [12]. Iron and its homeostasis play a critical role in the outcome of viral infections. As the virus relies on iron for replication, it tends to take this vital element out of the body’s physiological cycle and seize it for its survival [13]. Changes in the metabolism of this element in viral and also inflammatory diseases have also been studied and proven in several researches [14, 15]. Selenium, on the other hand, is a micromineral element whose role in changing the immune response pattern and increasing antiviral defense has been extensively studied [16, 17]. Research during the recent pandemic shows that the supplementation of this element reduces the risk and severity of COVID-19 [18].

Over the last decade, studies on the structures synthesized based on nanochelating technology have demonstrated the therapeutic effects of these medicines in cellular and animal models of various diseases [19–21]. Through intelligent modification of trace elements metabolism and related mechanisms, these medicines can induce immunomodulatory behavior and subsequent therapeutic effects [22, 23]. Previous studies have demonstrated the antioxidant, antineoplastic, and immunomodulatory effects of BCc1 nanomedicine (which has iron-chelating property) without causing any side effects in several cellular and animal models as well as clinical trials [21, 24].

Given the established role of iron and selenium metabolism in the immune system responses and outcomes of viral diseases, the current study aimed to assess the impact of adding the combination of BCc1 (having iron-chelating property) and Hep-S (selenium-containing) nanomedicines to the standard treatment of hospitalized COVID-19 patients.

## Methods

### Trial design

Eligible COVID-19 patients who were hospitalized at Masih Daneshvari Hospital in Tehran, Iran were enrolled in this randomized, hospital-based, parallel-group, placebo-controlled trial to evaluate the effects of the combination of BCc1 and Hep-S nanomedicines on moderate COVID-19 patients.

### Participants’ inclusion and exclusion criteria

Hospitalized confirmed COVID-19 patients, diagnosed via PCR and CT scanning of the lungs by WHO diagnosis criteria, were selected and recruited for the present study. All the patients filled out a consent form to participate in this study. Pregnant, lactating, inherited immunodeficiency, transplanted, and diabetes type 1 patients, as well as alcohol and drug consumers, were excluded from the trial.

### Study setting

The current study was performed and supervised by nurses and doctors at Masih Daneshvari Hospital. The comprehensive procedure of the trial was explained to the patients by the recruited nurses at the hospital and then an informed written consent form was signed by all the patients.

### Interventions

**Fig. 1:**
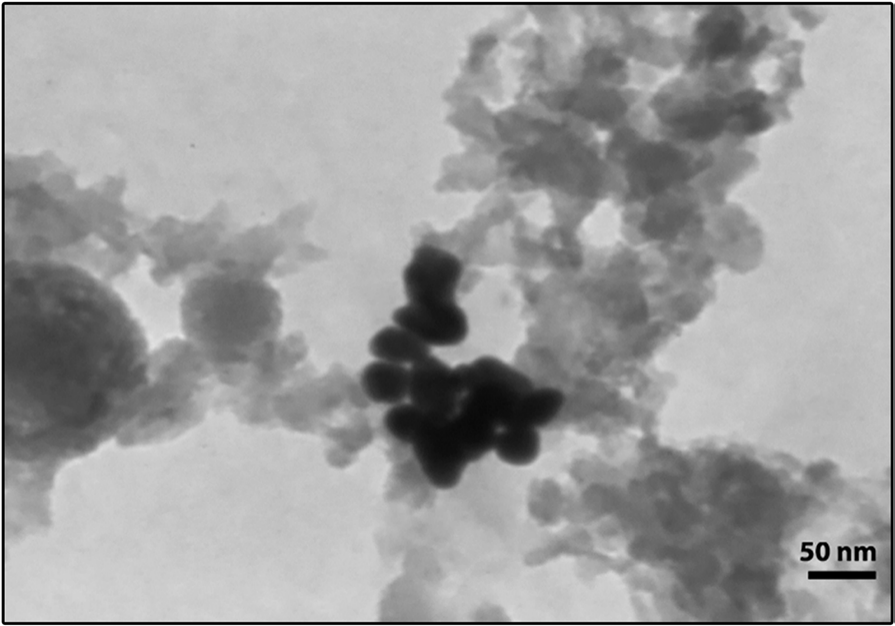
TEM image of Hep-s

BCc1 and Hep-S nanomedicines were designed by Sodour Ahrar Shargh Company based on nanochelating technology [25]. BCc1 characterization and its standard median lethal dose (LD50) are reported in previous studies [21, 26]. Hep-S is a selenium-containing nanochelating-based structure. The HRTEM image of Hep-S was captured using a Philips CM30-250kV model transmission electron microscope at the University of Tehran Science and Technology Park. The HRTEM image of Hep-S indicates that the size of this nanomedicine is approximately 22.7nm (Fig. 1).

Hep-S toxicity was evaluated based on the guidelines of the Organization for Economic Co-operation and Development (OECD, guideline 420) regulations and by the LD50 evaluation test; these tests were conducted in the School of Pharmacy at Tehran University of Medical Sciences. The toxicity report of Hep-S shows that i.p LD50 of this structure is 54 mg/kg. Active pharmaceutical ingredient (API) synthesis of BCc1 and Hep-S nanomedicines was carried out by using the nanochelating technology in the laboratory of Sodour Ahrar Shargh Company.

The two nanomedicines of BCc1 and Hep-S were used at the same time in the form of syrup as a two-medicine package to evaluate its effectiveness in comparison with a placebo. Two types of placebo syrup were administered to the patients in the placebo group. Both the COVID-19 and placebo syrup were identical in terms of shape and size.

Each nanomedicine was provided in a separate bottle along with instructions for each. The patients in the treatment group received BCc1 twice a day (1500 mg per serving) and Hep-S once a day (1500 μg per serving) for 28 days.

### Outcomes

IL6 level was defined as the primary outcome of the present study and clinical score was mentioned as the second outcome.

### Randomization, blinding, and allocation

All patients, clinicians, nurses, and researchers were blinded to the allocation of treatments. The patients were assigned to the study after the clinicians screened them based on the inclusion and exclusion criteria. They were then randomly assigned to the treatment or placebo group based on a block randomization form prepared and given to the nurses by the researcher in charge. All the patients signed an informed written consent form.

### Sample size

The sample size was determined by the number of eligible participants who agreed to participate in the study between October 2 and March 20, 2020, ensuring that the entire population of interest was allocated. So, 62 patients in the treatment and 60 patients in the placebo groups participated in the present study. The decision to use all available patients in the study was based on similar studies in the literature that investigated changes in cytokine levels over time and often employed a similar approach, utilizing the available participants who met the inclusion criteria [27, 28]. This allows for a more comprehensive analysis of the specific population under investigation.

### Withdrawal

At any point during the study, the patients were all allowed to withdraw from the experiment and were not asked to provide the reason, but in case of withdrawal, they allowed the continuation of data collection.

### Follow-up

During the hospitalization period, the medicines were administered to the patients by the nurses according to the clinicians’ prescriptions. The patients were followed up 24 h after being discharged from the hospital. They were also contacted on days 10, 15, 20, and 27 by the study team to assure that the patients had taken the medicines.

Besides, the patients had access to the researcher in charge by phone calls to consult with her for any reason at any time. Trial completion was defined as consuming the nanomedicines for 28 days or discontinuation of the follow-ups for any cause.

### Data collection

During the study, the researcher in charge collected the information and checked for any missing values and inconsistencies. Full details of the data collection procedure are available upon request.

### Assessments

- Blood samples were taken from all 122 patients in the treatment and placebo groups on day zero (before medicines consumption), at discharge, and 28 days after consumption (end of the treatment) to measure biochemical indices (Table 1). All tests were carried out in the clinical laboratory of Masih Daneshvari Hospital according to the standard protocols of the hospital.
- Eleven patients from each group (22 samples in total) were randomly selected to measure serum levels of INF-γ, TNF-α, and IL-6 cytokines before the start of the study, at discharge, and after the end of the treatment. ELISA kits were used to measure TNFα (R&D Systems, UK), IFNγ (Thermo Fisher, Waltham, Massachusetts, USA), and IL-6 (R&D Systems, UK) according to the manufacturer’s instructions [29].
- The clinical symptoms of all the patients were recorded according to an assessment questionnaire (Table 2) before the start of the treatment and on days 3, and discharge date, which was on day 6 or 7 of hospitalization. The responses were then scored with the highest number representing better health conditions. As this study was conducted on the third peak of COVID-19 disease (in autumn and winter, 2020–2021), the patients were discharged from the hospital as soon as their standard treatment period (remdesivir, etc.) was finished (day 6 or 7 of hospitalization) so that new COVID-19 patients could be hospitalized. Therefore, it was practically impossible to compare the hospitalization period of the patients in both groups.

**Table 1:** Titles of laboratory tests

	Laboratory features	Before medicine consumption	At discharge of hospital	28 days after consumption
1	AST	*	*	*
2	ALT	*	*	*
3	Ferritin	*	*	*
4	Total Bilirubin	*	*	*
5	Serum Iron	*	*	*
6	TIBC	*	*	*
7	ESR	*	*	*
8	CRP	*	*	*

**Table 2:** Titles of clinical score

Title	Not (2)	Sometimes (1)	Yes (0)
Headache			
Need to oxygen therapy			
Anosmia			
Cough			
Fatigue			

### Statistical analysis

Descriptive statistics were expressed using mean ± standard deviation (SD), median (Q1, Q3), and minimum–maximum. The mean difference of variables between the nanomedicines and placebo was evaluated using an independent *t*-test or Mann–Whitney *U* test. The repeated measure analysis was used to assess the impact of time and treatment on the markers. The post hoc analysis was performed between times using Bonferroni multiple comparisons. The estimated marginal means of markers are shown using a profile plot by time and treatment. The Wilcoxon signed ranks test was used to compare the markers at different times relative to the value of the marker in the baseline. All analyses were performed by R (version 4.0.2) and SPSS (version 26). *P*-values of less than 0.05 were regarded as statistically significant.

## Results

### Patients’ disposition and characteristics

**Fig. 2:**
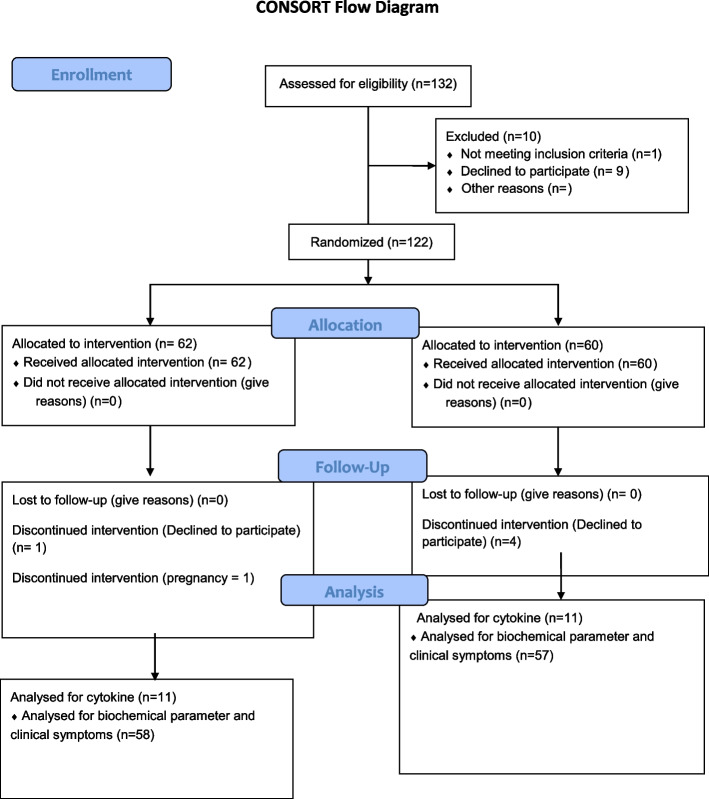
Consort flow diagram of study

The patients were recruited between Oct 2, 2020, and March 20, 2021. Initially, 132 patients were randomly recruited. Due to ineligibility, and incomplete histological confirmation, among other reasons, ten of those were excluded from the study (Fig. 2, prepared according to the consolidated Standards of Reporting Trials Form) [30]. The patient’s demographic information is shown in Table 3. All the patients received similar antiviral therapy, including remdesivir, dexamethasone, and prednisolone.

**Table 3:** Descriptive statistics of patients in the combination of BCc1 and Hep-S group with patients in the placebo group

Variable	Level	Nanomedicines (*N* = 62)	Placebo (*N* = 60)
Sex	Male	29 (44.6%)	37 (64.9%)
	Female	36 (55.4%)	20 (35.1%)
Age	Mean ± SD	50.65 ± 11.82	52.23 ± 13.46
	Median (IQR)	53.00 (39.50, 59.50)	42.00 (53.00, 61.00)
Difference time of discharge and hospitalization	Mean ± SD	6.92 ± 4.09	6.25 ± 1.71
	Median (IQR)	6.00 (5.00, 7.00)	6.00 (5.00, 7.00)
Difference time of discharge and taking intervention	Mean ± SD	5.78 ± 4.16	5.19 ± 1.59
	Median (IQR)	5.00 (4.00, 6.00)	5.00 (4.00, 6.00)

^a^The exact Pearson chi-square

^b^the independent *t*-test

^c^the exact Mann–Whitney test

### Outcome and estimation

#### Serum cytokine levels

**Fig. 3:**
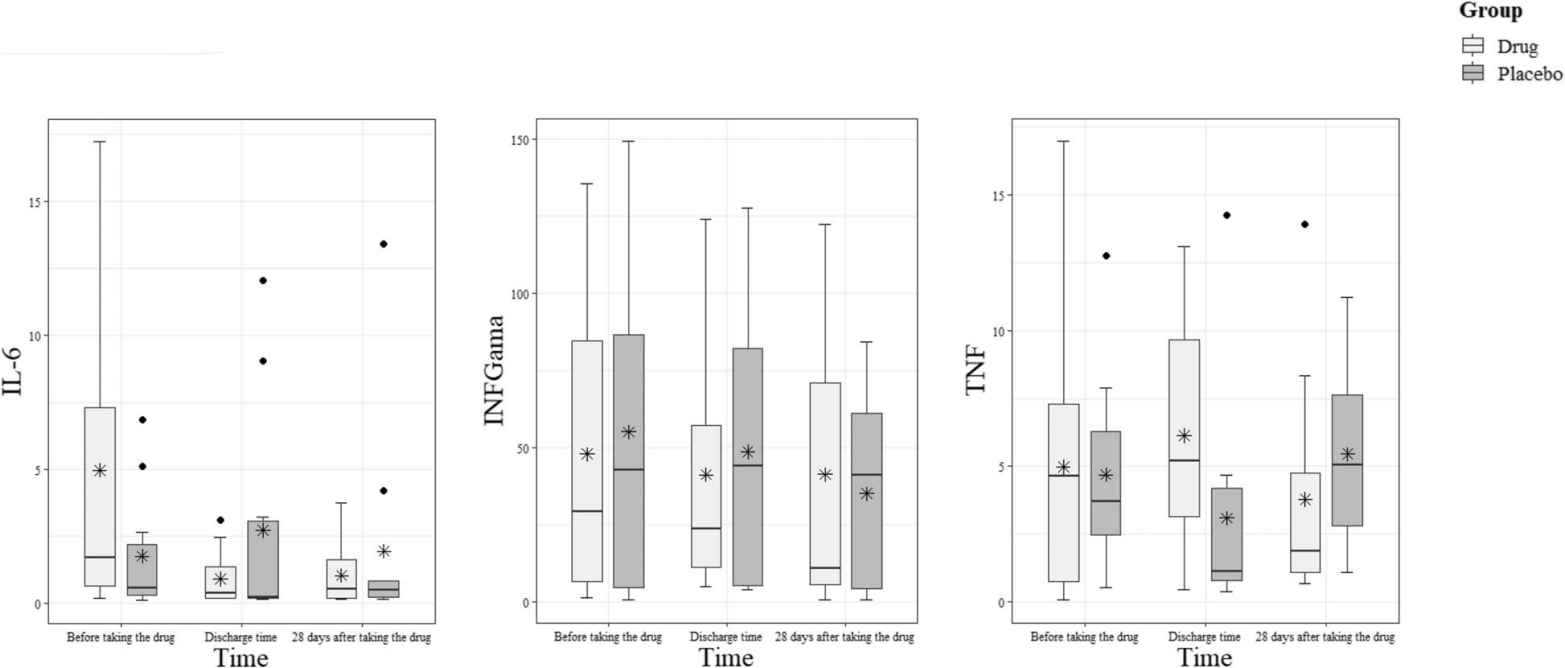
Comparison of dot plot diagram of three cytokines (IL6, TNFα, and TNFγ) of patients in the combination of BCc1 and Hep-S group with patients in the placebo group before medicine consumption, at discharge of hospital and 28 days after consumption

Measuring biomarkers before the start of the study, at discharge, and at the end of the treatment showed changes in their levels in the treatment group, especially the levels of IL-6 (Fig. 3 and Table 4).

**Table 4:** Descriptive statistics of cytokine tests by group (drug vs. placebo)

A. Mauchly’s test of sphericity for IL-6, TNF-α, and IFN-γ									
	Within Subjects Effect		Mauchly’s W	Approx. chi-square	df	Sig	Epsilon^b^		
							Greenhouse–Geisser	Huynh–Feldt	Lower-bound
IL-6	Time		.903	1.844	2	.398	.911	1.000	.500
TNF	Time		.710	6.508	2	.039	.775	.870	.500
INFGama	Time		.438	15.699	2	.000	.640	.699	.500
B. Tests of within-subjects effects									
Variable	Source	Correction	Type III sum of squares	df	Mean square	F	Sig	Partial eta squared	Observed power^a^
IL-6	Time	Sphericity Assumed	37.497	2	18.748	1.580	.219	.077	.314
	Time * Group	Sphericity Assumed	82.868	2	41.434	3.493	.041	.155	.617
	Error(Time)	Sphericity Assumed	450.792	38	11.863				
TNF	Time	Greenhouse–Geisser	.682	1.550	.440	.023	.953	.001	.053
	Time * Group	Greenhouse–Geisser	61.539	1.550	39.693	2.059	.153	.093	.347
	Error(Time)	Greenhouse–Geisser	597.626	31.007	19.274				
INFGama	Time	Greenhouse–Geisser	1945.471	1.280	1519.712	1.102	.321	.052	.188
	Time * Group	Greenhouse–Geisser	670.163	1.280	523.501	.380	.595	.019	.095
	Error(Time)	Greenhouse–Geisser	35297.486	25.603	1378.638				

IL-6: There was a significant main effect of time (*F* = 1.580, *p* = 0.219, partial eta squared = 0.077), indicating that IL-6 levels changed over time. Additionally, the interaction effect of time and group was significant (*F* = 3.493,* p* = 0.041, partial eta squared = 0.155), suggesting that the change in IL-6 levels differed between the treatment and placebo groups. Numerically, there was a 77% downward trend in IL-6 during the nanomedicine consumption and an 18% increase in the placebo group.

TNF: The main effect of time was not significant (*F* = 0.440, *p* = 0.953), indicating no significant change in TNF levels over time. The interaction effect of time and group was also not significant (*F* = 2.059,* p* = 0.153), suggesting no differential impact of the treatment on TNF levels between the groups. Numerically, there was a 21% decrease in TNF-α cytokine level in the treatment group, while there was a 31% increase in the level of this cytokine in the placebo.

INF-Gama: Similar to TNF, there was no significant main effect of time (*F* = 1.102, *p* = 0.321) or interaction effect of time and group (*F* = 0.380, *p* = 0.595) for INF-Gama levels. This implies that there were no significant changes in INF-Gama levels over time and no differential impact of the treatment between the groups.

### Biochemical parameters

The results indicated that all the measured biological and laboratory parameters according to Table 1 were at normal range on day 28, and there was no significant difference between the treatment and placebo groups (Table 5).

**Table 5:** (A) Descriptive statistics of biochemical tests by group (nanomedicines vs. placebo). (B) Tests of within-subjects effects

A										
**Variables**	**Time point**	**Group**	**Mean ± SD**		**Median (Q1, Q3)**			**Min, Max**		***P*-value**
Ferritin	Before taking the Nanomedicines	Nanomedicines	467.83 ± 482.72		338.00 (162.50, 564.50)			4.00, 2000.00		0.690
		Placebo	505.00 ± 355.59		407.00 (236.00, 739.00)			36.00, 1660.00		
	Time of discharge	Nanomedicines	425.70 ± 356.34		421.00 (51.25, 672.00)			27.00, 1105.00		0.837
		Placebo	391.20 ± 382.63		248.00 (120.00, 580.25)			47.00, 1189.00		
	28 days after taking the Nanomedicines	Nanomedicines	241.37 ± 203.53		196.50 (83.00, 349.75)			6.00, 762.00		0.961
		Placebo	243.85 ± 200.92		224.00 (67.00, 348.00)			20.00, 737.00		
AST	Before taking the Nanomedicines	Nanomedicines	44.28 ± 18.27		40.00 (30.00, 56.00)			14.00, 117.00		0.366
		Placebo	47.63 ± 23.28		41.00 (31.25, 55.00)			15.00, 127.00		
	Time of discharge	Nanomedicines	56.89 ± 49.51		43.50 (32.25, 60.50)			15.00, 341.00		0.303
		Placebo	49.53 ± 22.12		47.00 (28.50, 60.50)			18.00, 106.00		
	28 days after taking the Nanomedicines	Nanomedicines	28.38 ± 14.89		25.00 (21.00, 31.25)			13.00, 112.00		0.808
		Placebo	29.07 ± 12.41		25.00 (20.75, 34.50)			15.00, 69.00		
ALT	Before taking the Nanomedicines	Nanomedicines	42.04 ± 24.37		35.00 (28.00, 48.00)			9.00, 131.00		0.025
		Placebo	53.72 ± 32.58		41.50 (30.25, 71.00)			11.00, 191.00		
	Time of discharge	Nanomedicines	86.70 ± 63.93		63.00 (41.00, 117.25)			22.00, 416.00		0.203
		Placebo	100.72 ± 55.65		90.00 (59.00, 129.00)			20.00, 252.00		
	28 days after taking the Nanomedicines	Nanomedicines	38.96 ± 28.54		31.00 (24.00, 42.50)			4.00, 156.00		0.466
		Placebo	43.35 ± 29.92		36.50 (26.50, 50.50)			12.00, 160.00		
Bill.Total	Before taking the Nanomedicines	Nanomedicines	0.62 ± 0.78		0.50 (0.30, 0.70)			0.10, 6.50		0.717
		Placebo	0.58 ± 0.24		0.60 (0.40, 0.70)			0.20, 1.30		
	Time of discharge	Nanomedicines	0.57 ± 0.32		0.50 (0.40, 0.80)			0.10, 2.00		0.401
		Placebo	0.62 ± 0.33		0.60 (0.40, 0.80)			0.04, 2.00		
	28 days after taking the Nanomedicines	Nanomedicines	0.92 ± 0.48		0.80 (0.60, 1.13)			0.20, 2.70		0.927
		Placebo	0.93 ± 0.53		0.70 (0.60, 1.20)			0.40, 2.70		
IRON.SEROM	Before taking the Nanomedicines	Nanomedicines	56.16 ± 24.18		51.75 (42.25, 69.75)			18.00, 124.00		0.391
		Placebo	61.28 ± 25.66		51.20 (43.00, 81.00)			25.00, 125.90		
	Time of discharge	Nanomedicines	88.89 ± 36.83		80.00 (62.00, 124.00)			29.70, 159.00		0.115
		Placebo	102.71 ± 41.60		98.60 (75.40, 129.00)			30.00, 258.00		
	28 days after taking the Nanomedicines	Nanomedicines	82.94 ± 32.33		81.00 (59.45, 108.53)			20.00, 152.00		0.820
		Placebo	81.44 ± 26.37		76.00 (68.00, 94.00)			24.00, 155.00		
TIBC	Before taking the Nanomedicines	Nanomedicines	225.31 ± 57.95		211.00 (188.00, 241.00)			142.00, 411.00		0.097
		Placebo	207.23 ± 30.05		208.00 (186.00, 224.00)			152.00, 277.00		
	Time of discharge	Nanomedicines	252.05 ± 61.07		249.00 (199.00, 302.00)			137.00, 380.00		0.013
		Placebo	222.29 ± 36.61		224.00 (200.25, 242.75)			136.00, 341.00		
	28 days after taking the Nanomedicines	Nanomedicines	291.79 ± 61.10		283.00 (246.00, 318.00)			191.00, 485.00		0.441
		Placebo	280.92 ± 63.75		270.50 (238.50, 333.25)			80.70, 418.00		
CRP	Before taking the Nanomedicines	Nanomedicines	43.52 ± 24.71		43.00 (23.00, 62.00)			1.00, 100.00		0.111
		Placebo	50.50 ± 24.21		51.00 (31.00, 73.50)			1.00, 95.00		
	Time of discharge	Nanomedicines	10.55 ± 10.44		8.00 (4.25, 12.75)			1.00, 63.00		0.635
		Placebo	11.58 ± 13.27		7.00 (3.00, 15.00)			1.00, 68.00		
	28 days after taking the Nanomedicines	Nanomedicines	8.80 ± 11.82		4.00 (1.00, 10.00)			1.00, 51.00		0.328
		Placebo	11.48 ± 13.70		6.00 (2.00, 15.75)			1.00, 52.00		
ESR	Before taking the Nanomedicines	Nanomedicines	46.16 ± 28.31		42.00 (22.00, 66.00)			1.00, 102.00		0.749
		Placebo	47.70 ± 25.51		48.00 (27.25, 65.00)			4.20, 120.00		
	Time of discharge	Nanomedicines	18.94 ± 17.50		14.50 (5.25, 25.75)			1.00, 79.00		0.870
		Placebo	18.41 ± 17.70		12.00 (5.00, 24.00)			2.00, 86.00		
	28 days after taking the Nanomedicines	Nanomedicines	19.82 ± 17.21		14.00 (7.75, 27.50)			2.00, 65.00		0.545
		Placebo	22.06 ± 19.20		14.00 (9.00, 32.00)			2.00, 76.00		
B										
Variable	Source		Type III sum of squares	df	Mean square	F	Sig	Partial eta squared	Noncent. parameter	Observed power^a^
Ferritin	Time	Sphericity Assumed	183,646.752	2	91,823.376	1.732	.226	.257	3.465	.281
Ferritin	Time * Group1	Sphericity Assumed	1397.610	2	698.805	.013	.987	.003	.026	.051
Ferritin	Error(Time)	Sphericity Assumed	530,063.533	10	53,006.353					
AST	Time	Greenhouse–Geisser	27,066.771	1.788	15,138.097	31.472	.000	.255	56.271	1.000
AST	Time * Group1	Greenhouse–Geisser	511.636	1.788	286.152	.595	.535	.006	1.064	.142
AST	Error(Time)	Greenhouse–Geisser	79,122.678	164.495	481.003					
ALT	Time	Greenhouse–Geisser	150,147.050	1.553	96,703.075	48.829	.000	.347	75.815	1.000
ALT	Time * Group1	Greenhouse–Geisser	910.287	1.553	586.276	.296	.688	.003	.460	.091
ALT	Error(Time)	Greenhouse–Geisser	282,895.072	142.845	1980.437					
Bill.Total	Time	Greenhouse–Geisser	6.072	1.721	3.528	11.316	.000	.133	19.478	.984
Bill.Total	Time * Group1	Greenhouse–Geisser	.152	1.721	.088	.283	.720	.004	.488	.091
Bill.Total	Error(Time)	Greenhouse–Geisser	39.708	127.370	.312					
IRON.SEROM	Time	Sphericity Assumed	29,250.208	2	14,625.104	14.624	.000	.230	29.249	.999
IRON.SEROM	Time * Group1	Sphericity Assumed	1598.228	2	799.114	.799	.453	.016	1.598	.183
IRON.SEROM	Error(Time)	Sphericity Assumed	98,004.157	98	1000.042					
TIBC	Time	Greenhouse–Geisser	65,970.480	1.648	40,026.962	12.705	.000	.213	20.939	.989
TIBC	Time * Group1	Greenhouse–Geisser	6333.501	1.648	3842.791	1.220	.295	.025	2.010	.237
TIBC	Error(Time)	Greenhouse–Geisser	244,052.483	77.463	3150.564					
CRP	Time * Group1	Greenhouse–Geisser	917.975	1.543	595.113	1.631	.204	.019	2.516	.298
CRP	Time * Group1	Greenhouse–Geisser	823.985	1.623	653.123					
CRP	Error(Time)	Greenhouse–Geisser	47,842.735	131.114	364.893					
ESR	Time	Greenhouse–Geisser	47,887.202	1.699	28,178.876	72.825	.000	.439	123.758	1.000
ESR	Time * Group1	Greenhouse–Geisser	.658	1.699	.387	.001	.997	.000	.002	.050
ESR	Error(Time)	Greenhouse–Geisser	61,153.856	158.044	386.941					

### Clinical score

**Fig. 4:**
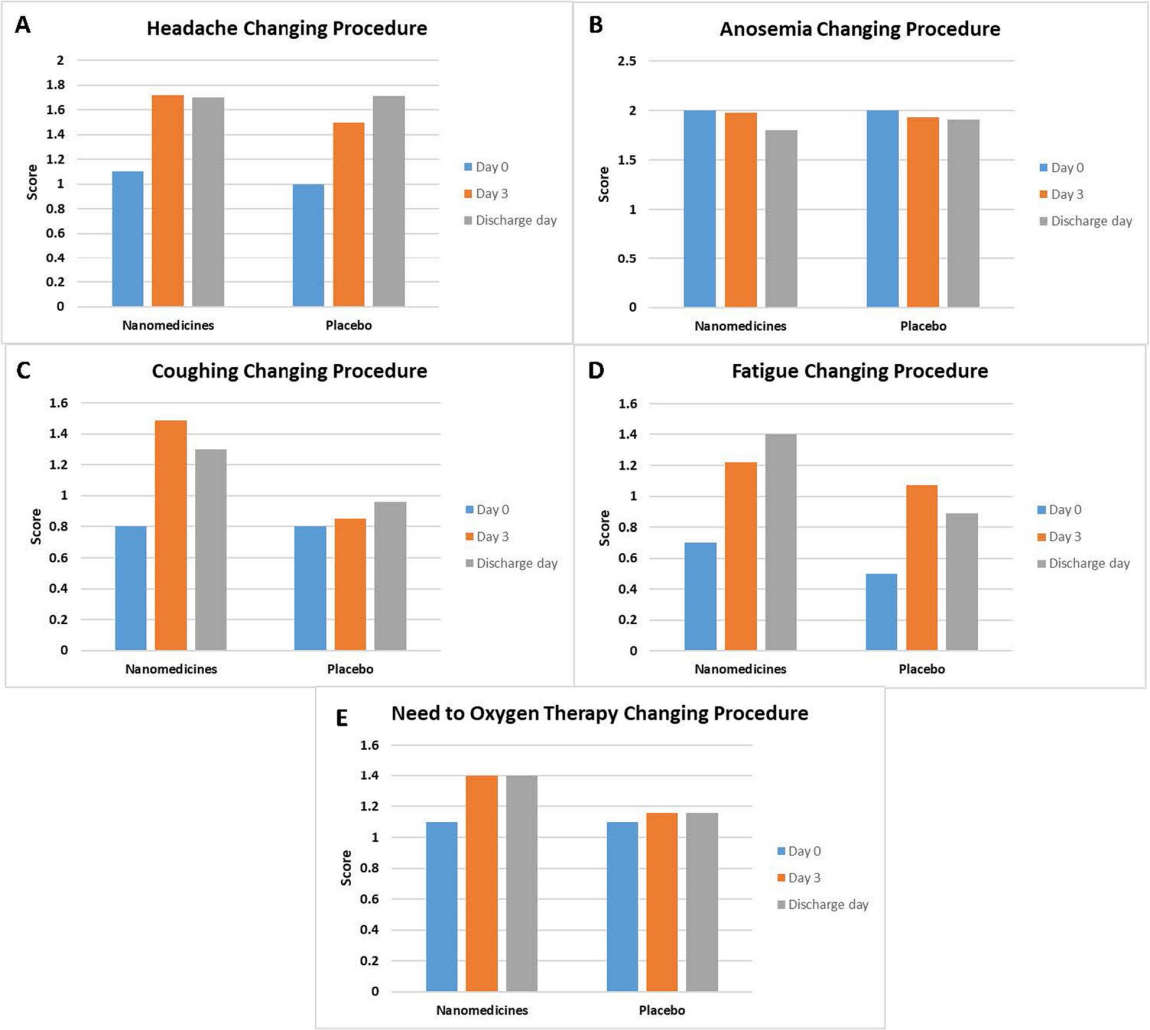
Clinical score in placebo and nanomedicines-treated groups. The higher number represents better health conditions. The clinical score evaluation showed that the mean score of coughing, fatigue, and need for oxygen therapy on discharge day in the nanomedicines-treated group improved more than the placebo group

All patients’ clinical symptoms were documented using an assessment questionnaire (Table 2) before the start of therapy, on days 3 and on their discharge date, which was on days 6 or 7 of hospitalization. The responses were then scored, with the highest score indicating the highest health condition. The clinical score evaluation showed that the mean score of cough and fatigue on discharge day in the nanomedicines-treated group improved by 60% and 100%, respectively, compared to day 0; however, in the placebo group, these parameters increased by 20% and 78%. In addition, the mean score of need for oxygen therapy on discharge day in the nanomedicines-treated group ameliorated by 27% while it was 5% in the placebo group (Fig. 4A–E).

### Survival

The results of the statistical analyses showed two and three death cases in the treatment and placebo groups, respectively. The comparisons also indicated that the deceased patients in the treatment group lived 4 days longer than the deceased in the placebo group, but the change was not significant (Table 6).

**Table 6:** Statistical analyses of death cases in the treatment and placebo groups

	**Group**	***N***	**Mean**	**Std. deviation**	**Std. error mean**
Time	4, 5	2	15.0000	4.24264	3.00000
	6, 7	3	11.0000	5.19615	3.00000

## Discussion

CCS is a pathological and systemic inflammatory syndrome involving increased levels of circulating cytokines and immune-cell hyperactivation which can be triggered by various therapies, pathogens, cancers, autoimmune conditions, etc.

The clinical signs of COVID-19 range greatly, from moderate to severe cases of atypical pneumonia, with some developing acute respiratory distress syndrome (ARDS), which frequently necessitates invasive mechanical ventilation and is the major cause of mortality. The severity of the respiratory disease caused by SARS-CoV-2 is thought to be largely owing to an increased immunological response to the virus and CCS [5, 31–34].

Oxidative stress is a hallmark of inflammation and COVID-19 disease, which is connected to the CSS seen in patients with severe COVID-19 [35, 36]. Selenium is essential to boost immunity, lower oxidative stress, and prevent viral infections, resulting in the amelioration of severe diseases [18]. As a result, selenium supplementation can be used as a supportive treatment for COVID-19 infection, and various researchers have therefore looked into a justification for randomized, controlled trials of selenium supplementation in the disease caused by SARS-CoV-2 [18, 37, 38].

In viral infections, changes occur in the body’s iron metabolism aiming to seize iron and limit the virus’s access to this vital metal. However, these events, which are centered on proinflammatory cytokines including IL-6, lead to altered iron metabolism and increased oxidative stress via the Fenton reaction, which results in ferroptosis and the continuation of oxidative harm to biomolecules that finally damage the organs in the body [39, 40].

Inflammation, oxidative stress, and altered iron homeostasis are inextricably connected at a systemic level [41].This viewpoint emphasizes the possible role of altered iron homeostasis as well as its potential significance in viral diseases’ pathogenesis and management strategies [42, 43]. Surprisingly, in the natural immunity of the body, there are iron chelators whose antiviral effects are proven in numerous studies. Lactoferrin (Lf) is a widely distributed glycoprotein generated by a variety of mucosal epithelial cells and is an important component of natural immunity. This protein can chelate iron and its antiviral capacity is demonstrated in previous studies [44], and even several researches have discussed its potential for antiviral therapy.

As a result, given the vital role of this element for hemoglobin synthesis and other physiological processes, iron chelation therapy can be used as a strategy for managing iron dis-homeostasis with the aim of iron redistribution and sequestration to make iron inaccessible to viruses, while preventing its excretion. It should be noted that the existing iron chelators have many limitations making them incapable of such smart therapeutic behavior. Among the existing chelators, deferiprone has shown higher capability to redistribute iron in various experiments [45, 46]. However, although the existing iron chelators have demonstrated promising impacts on viral diseases—mostly in vitro and rarely clinical studies—they are not yet nominated as serious operational candidates for the treatment of viral diseases due to their side effects and structural limitations. These limitations are to such an extent—even in their specific field of application (i.e., iron excretion in diseases caused by iron overload)—that there is a serious need for more efficient chelators [47].

In the previous studies, we reported the successful effects of BCc1 nanochelating-based iron chelator in animal and clinical studies. This nanomedicine increased the survival and quality of life of metastatic and non-metastatic gastric cancer patients without any side effects [21, 48] and showed nephroprotective and antioxidative effects in the animal model of chronic kidney disease [22].

**Fig. 5:**
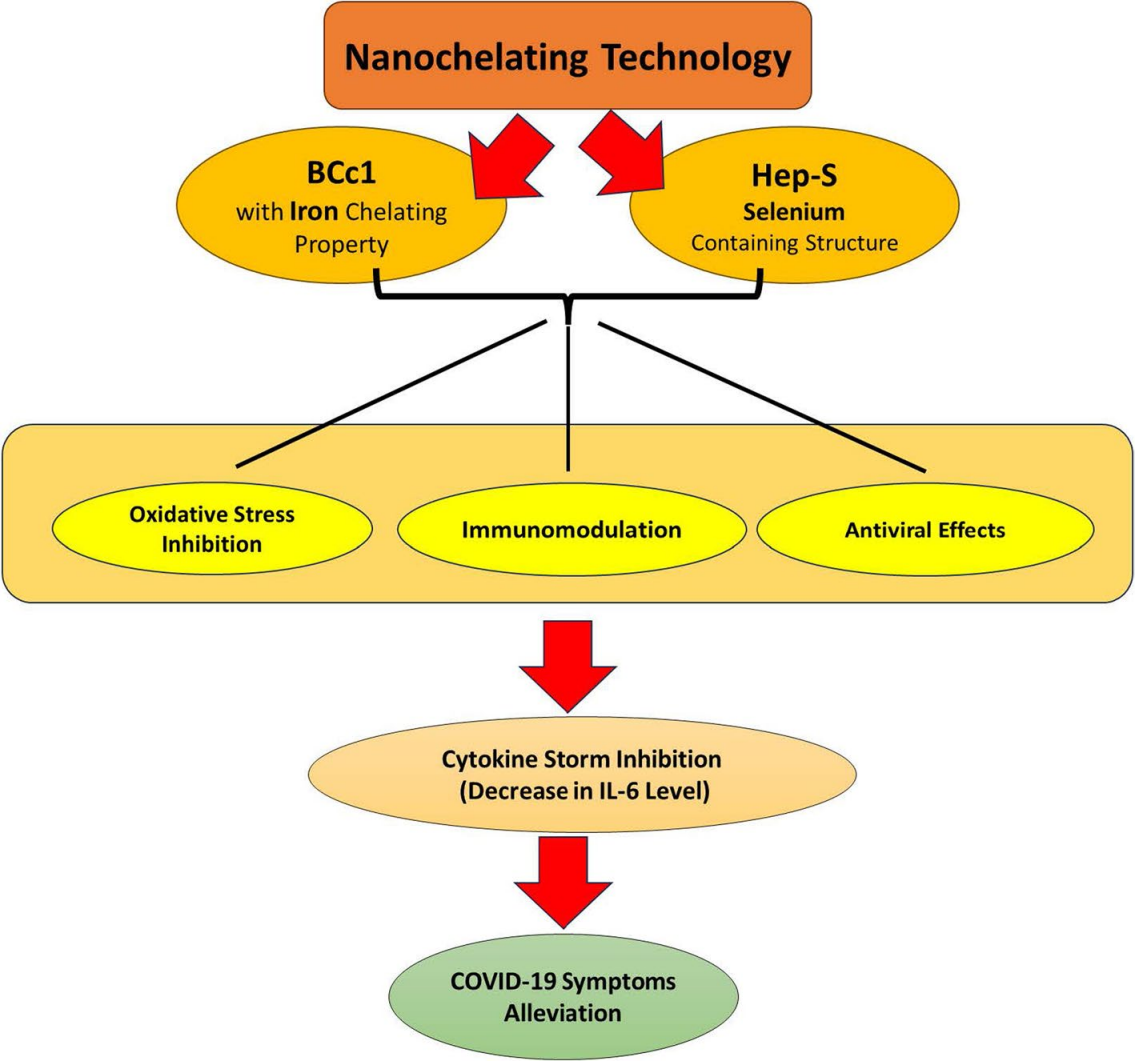
The schema of the proposed mechanism for the inhibitory effects of combination therapy by BCc1 and Hep-S nanomedicines on COVID-19. According to the previous studies conducted on BCc1 nanomedicine and the well-known role of selenium and iron elements on inflammatory cytokines level regulation, the immune system response against viral infection and the activity of antioxidant enzymes, the above pathways are suggested as effective mechanisms in combination therapy by these nanomedicines

Given the proven impact of iron and selenium on the immune system and in light of the results of the previously reported study on BCc1 nanomedicine (Fig. 5), the effect of the combination of BCc1 and selenium-containing Hep-S nanomedicines on hospitalized COVID-19 patients were evaluated in the current study.

The results showed that adding the combination therapy of BCc1 and Hep-S nanomedicines to the standard treatment of hospitalized COVID-19 patients had no negative effect on their hematological and biochemical parameters. As explained in the “Results” section, the characteristics linked to the physiological function of iron, such as hemoglobin, red blood cell count, and hematocrit, were assessed in this study, and the results showed that despite the iron-chelating property of BCc1, the combination therapy of BCc1 and Hep-S had no negative impact on the indices. The results of this study were in line with the results of the study on gastric cancer patients conducted by Hafizi et al., demonstrating that the 18-month consumption of BCc1 nanomedicine had no negative effect on hematological indices compared to the placebo group [21].

Studies have reported an increase in the plasma levels of IL-6 and TNF-alpha in hospitalized COVID-19 patients [49]. The higher level of IL-6 concentration is closely related to the requirement for ventilatory assistance and the development of respiratory failure [50]. Suppressing this cytokine, therefore, results in managing clinical symptoms, shortening the hospitalization period, and decreasing the need for oxygen therapy [51]. According to the CSS pathogenesis in COVID-19, immunomodulatory therapy can be a proper consideration in this disease [52]. Immunomodulatory medications, which operate by modifying or harnessing the immune responses, come with several disadvantages and side effects that can negatively impact patients’ quality of life. Unwanted side effects, such as severe infections, cytokine release syndrome, anaphylaxis, and hypersensitivity as well as immunogenicity, make developing novel and safer immunomodulatory structures difficult [53, 54].

Since IL-6 is a relevant cytokine in acute respiratory distress syndrome, the blockade of its receptor with tocilizumab (TCZ) in COVID-19 patients has been evaluated in numerous studies. Some showed the beneficial effect of this medicine on reducing mortality rate and hospitalization time [55], while several experiments showed its failure [56, 57] and even did not support its use for the management of cytokine storm in COVID-19 patients [58]. Also, several studies reported that the incidence of infectious complications in patients receiving TCZ was higher than in patients receiving standard therapies [55].

In the present study, consuming the combination of BCc1 and Hep-S nanomedicines reduced IL-6 cytokine significantly and could also reduce the numeral value of TNF-α. In addition, the comparison of the treatment and placebo groups showed that these two nanomedicines could decrease the IL-6/IFN-γ ratio; the higher this ratio is, the more serious the CSS and damage to the lungs will be [59].

According to studies on the prevalence of clinical symptoms of COVID-19, cough, fatigue, fever, and dyspnea are the most common hallmarks in COVID-19 patients [60]. The results of the patients who received nanomedicine in the current study showed decreased fatigue, coughing, and the need for oxygen therapy. In the study by Larvie et al., selenium consumption was shown to be inversely associated with the severity of COVID-19 symptoms, emphasizing the relationship between selenium consumption and the inflammatory response in COVID-19 patients [61]. Researchers found that altering metal element metabolisms, such as selenium and iron, can interrupt the infectious relationship between the virus and the host, alleviating COVID-19 symptoms [62, 63]. One reason for the immunomodulatory effect of these two nanomedicines, without causing any abnormal changes in blood haemato- and biochemical parameters or clinical symptoms, etc. during consumption, is their smart impact on the metabolism of two vital elements of iron and selenium by benefiting from their unique high-tech structure. These effects of nanomedicines on clinical symptoms can be evaluated in larger studies to show the repeatability of the results.

Studies show that iron chelation exhibits antiviral and immunomodulatory effects in vitro [64] and in vivo, can attenuate ARDS and help control SARS-CoV-2 [42]. In addition, there is a risk of selenium insufficiency in immunopathological conditions, and as a result of this, blood selenium levels are more likely to decline. According to studies, serum IL-6 concentrations are inversely linked to serum selenium [65, 66]. Selenium-deficient cells generate more IL-6 in human bronchial epithelial cell lines infected with influenza virus [67]. There is also evidence that selenium supplementation can reduce excessive cytokine production [68].

Previous studies on nanochelating-based structures have evaluated and proved the immunomodulatory effects of these structures. In an animal model of multiple sclerosis as an autoimmune disorder, Fakharzadeh et al. showed that MSc1 nanochelating-based iron chelator could prompt therapeutic behavior, improve the disabling features of experimental autoimmune encephalomyelitis, and decrease lymphocyte infiltration in the central nervous system [23]. In another study, selenium and zinc-containing DIBc metal–organic framework demonstrated antidiabetic effects and lowered TNF-α levels efficiently [69].

Thus, it seems that the nanochelating technology has presented a new generation of immunomodulators with unique structures that do not suffer from limitations such as adverse reactions.

## Conclusion

The present study showed that the combination of BCc1 and Hep-S nanomedicines along with the standard treatments of COVID-19 reduced IL-6 as an important mediator of CSS and can be studied and evaluated in future clinical phases to present a novel immunomodulator.

### Supplementary Information

**Additional file 1:** **Table S** A) Descriptive Statistics of cell blood count by Group (nanomedicines vs. Placebo). B) Tests of Within-Subjects Effects. The blood samples were taken and analyzed on day zero, at discharge, and at the end of the treatment (on day 28). The results indicated that all the measured parameters were at normal range on day 28, and there was no significant difference between the treatment and placebo groups.

## Data Availability

The datasets generated during the current study are available from the corresponding author on reasonable request.
