# Supplementary material for: Beneficial effects of the combination of BCc1 and Hep-S nanochelating-based medicines on IL-6 in hospitalized moderate COVID-19 adult patients: a randomized, double-blind, placebo-controlled clinical trial
Source: Trials. 2023 Nov 11;24:720. doi: 10.1186/s13063-023-07624-2 (PMC10638761; doi:10.1186/s13063-023-07624-2)
Supplement: Supplementary file 1 — Additional file 1: Table S A) Descriptive Statistics of cell blood count by Group (nanomedicines vs. Placebo). B) Tests of Within-Subjects Effects. The blood samples were taken and analyzed on day zero, at discharge, and at the end of the treatment (on day 28). The results indicated that all the measured parameters were at normal range on day 28, and there was no significant difference between the treatment and placebo groups. [file 13063_2023_7624_MOESM1_ESM.docx]

Supplementary Table 1

1. Descriptive statistics of cell blood count by group (nanomedicines vs. placebo)

| **Variables** | **Time point** | **Group** | **Mean ± SD** | **Median (Q1, Q3)** | **Min, Max** | **P-value** |
| --- | --- | --- | --- | --- | --- | --- |
| HB | Before taking the Nanomedicines | Nanomedicines | 13.33±1.85 | 13.40 (12.50, 14.60) | 8.00, 17.00 | 0.137 |
|  |  | Placebo | 13.79±1.64 | 13.90 (12.50, 15.08) | 10.10, 17.80 |  |
|  | Time of discharge | Nanomedicines | 14.23±1.91 | 14.60 (13.08, 15.38) | 9.20, 18.20 | 0.484 |
|  |  | Placebo | 14.46±1.61 | 14.70 (13.40, 15.40) | 10.40, 17.50 |  |
|  | 28 days after taking the Nanomedicines | Nanomedicines | 13.92±1.72 | 13.90 (12.85, 14.95) | 7.70, 17.40 | 0.729 |
|  |  | Placebo | 13.79±1.88 | 14.00 (13.10, 15.00) | 5.85, 17.10 |  |
| RBC | Before taking the Nanomedicines | Nanomedicines | 4.71±0.48 | 4.70 (4.40, 5.12) | 3.04, 5.52 | 0.006 |
|  |  | Placebo | 4.98±0.58 | 4.99 (4.56, 5.25) | 3.45, 7.04 |  |
|  | Time of discharge | Nanomedicines | 5.04±0.65 | 4.91 (4.74, 5.29) | 3.79, 8.58 | 0.170 |
|  |  | Placebo | 5.22±0.75 | 5.23 (4.74, 5.62) | 3.01, 7.36 |  |
|  | 28 days after taking the Nanomedicines | Nanomedicines | 4.85±0.48 | 4.87 (4.60, 5.23) | 3.12, 5.75 | 0.188 |
|  |  | Placebo | 4.99±0.58 | 4.94 (4.63, 5.29) | 4.05, 7.17 |  |
| HCT | Before taking the Nanomedicines | Nanomedicines | 39.05±4.26 | 39.70 (36.60, 42.30) | 26.40, 47.00 | 0.095 |
|  |  | Placebo | 40.24±3.64 | 41.05 (37.55, 42.95) | 29.90, 47.60 |  |
|  | Time of discharge | Nanomedicines | 41.31±4.21 | 41.85 (39.53, 43.15) | 31.40, 50.00 | 0.155 |
|  |  | Placebo | 42.46±4.58 | 43.10 (40.30, 44.75) | 29.60, 56.40 |  |
|  | 28 days after taking the Nanomedicines | Nanomedicines | 41.23±4.61 | 42.00 (38.60, 44.55) | 24.40, 48.60 | 0.537 |
|  |  | Placebo | 41.76±3.62 | 41.90 (39.60, 44.50) | 32.70, 50.30 |  |
| MCV | Before taking the Nanomedicines | Nanomedicines | 83.07±6.39 | 84.50 (81.40, 87.10) | 57.80, 92.10 | 0.178 |
|  |  | Placebo | 81.36±7.80 | 83.00 (80.00, 85.80) | 56.80, 94.20 |  |
|  | Time of discharge | Nanomedicines | 83.03±6.45 | 84.20 (81.20, 86.50) | 60.00, 94.10 | 0.272 |
|  |  | Placebo | 81.56±8.05 | 82.85 (79.33, 85.78) | 56.90, 98.30 |  |
|  | 28 days after taking the Nanomedicines | Nanomedicines | 83.91±9.15 | 85.55 (81.48, 89.68) | 50.80, 95.00 | 0.837 |
|  |  | Placebo | 84.27±7.83 | 85.70 (82.10, 88.70) | 60.40, 96.50 |  |
| WBC | Before taking the Nanomedicines | Nanomedicines | 7.55±3.63 | 6.80 (4.90, 8.50) | 2.24, 22.20 | 0.308 |
|  |  | Placebo | 8.23±3.89 | 7.15 (5.50, 10.23) | 2.70, 18.90 |  |
|  | Time of discharge | Nanomedicines | 10.47±4.41 | 9.65 (7.70, 12.25) | 4.10, 26.60 | 0.673 |
|  |  | Placebo | 10.78±3.46 | 10.80 (8.20, 12.48) | 4.54, 20.30 |  |
|  | 28 days after taking the Nanomedicines | Nanomedicines | 8.02±2.61 | 7.79 (5.77, 9.28) | 3.38, 14.00 | 0.638 |
|  |  | Placebo | 7.78±2.41 | 7.28 (6.02, 9.40) | 3.04, 15.32 |  |
| Lymph | Before taking the Nanomedicines | Nanomedicines | 18.99±8.77 | 17.00 (13.20, 24.00) | 5.00, 45.00 | 0.050 |
|  |  | Placebo | 15.99±8.25 | 14.00 (9.78, 21.50) | 2.00, 39.00 |  |
|  | Time of discharge | Nanomedicines | 16.51±10.21 | 14.00 (11.08, 18.30) | 5.50, 57.00 | 0.090 |
|  |  | Placebo | 13.30±10.34 | 12.00 (8.13, 15.95) | 1.00, 81.00 |  |
|  | 28 days after taking the Nanomedicines | Nanomedicines | 30.64±10.96 | 29.85 (25.88, 39.48) | 0.21, 47.80 | 0.903 |
|  |  | Placebo | 30.39±9.47 | 28.65 (25.10, 35.90) | 0.28, 52.00 |  |
| Neut | Before taking the Nanomedicines | Nanomedicines | 74.69±11.16 | 77.00 (67.60, 83.00) | 46.00, 93.00 | 0.027 |
|  |  | Placebo | 78.95±10.11 | 81.00 (70.25, 87.00) | 49.97, 94.00 |  |
|  | Time of discharge | Nanomedicines | 78.02±10.49 | 81.40 (74.85, 84.78) | 36.00, 89.70 | 0.262 |
|  |  | Placebo | 80.26±11.25 | 81.50 (76.23, 85.30) | 16.00, 95.00 |  |
|  | 28 days after taking the Nanomedicines | Nanomedicines | 57.06±11.49 | 56.80 (49.33, 63.33) | 34.00, 92.00 | 0.844 |
|  |  | Placebo | 56.63±9.59 | 57.70 (49.50, 63.00) | 36.00, 74.40 |  |

1. Tests of Within-Subjects Effects

| Variable | Source | | Type III Sum of Squares | df | Mean Square | F | Sig. | Partial Eta Squared | Noncent. Parameter | Observed Power^a^ |
| --- | --- | --- | --- | --- | --- | --- | --- | --- | --- | --- |
| HB | Time | Greenhouse-Geisser | 24.665 | 1.777 | 13.882 | 12.693 | .000 | .121 | 22.552 | .993 |
| HB | Time * Group1 | Greenhouse-Geisser | 3.164 | 1.777 | 1.781 | 1.628 | .202 | .017 | 2.893 | .321 |
| HB | Error(Time) | Greenhouse-Geisser | 178.778 | 163.459 | 1.094 |  |  |  |  |  |
| RBC | Time | Greenhouse-Geisser | 3.995 | 1.874 | 2.132 | 16.616 | .000 | .150 | 31.139 | .999 |
| RBC | Time * Group1 | Greenhouse-Geisser | .230 | 1.874 | .123 | .955 | .382 | .010 | 1.790 | .208 |
| RBC | Error(Time) | Greenhouse-Geisser | 22.599 | 176.159 | .128 |  |  |  |  |  |
| HCT | Time | Sphericity Assumed | 218.386 | 2 | 109.193 | 18.948 | .000 | .169 | 37.895 | 1.000 |
| HCT | Time * Group1 | Sphericity Assumed | 6.436 | 2 | 3.218 | .558 | .573 | .006 | 1.117 | .142 |
| HCT | Error(Time) | Sphericity Assumed | 1071.904 | 186 | 5.763 |  |  |  |  |  |
| MCV | Time | Greenhouse-Geisser | 250.180 | 1.376 | 181.793 | 13.127 | .000 | .125 | 18.065 | .982 |
| MCV | Time * Group1 | Greenhouse-Geisser | 52.034 | 1.376 | 37.810 | 2.730 | .088 | .029 | 3.757 | .439 |
| MCV | Error(Time) | Greenhouse-Geisser | 1753.428 | 126.609 | 13.849 |  |  |  |  |  |
| WBC | Time | Sphericity Assumed | 471.659 | 2 | 235.829 | 32.899 | .000 | .259 | 65.797 | 1.000 |
| WBC | Time * Group1 | Sphericity Assumed | 12.490 | 2 | 6.245 | .871 | .420 | .009 | 1.742 | .198 |
| WBC | Error(Time) | Sphericity Assumed | 1347.652 | 188 | 7.168 |  |  |  |  |  |
| Lymph | Time | Greenhouse-Geisser | 13103.940 | 1.717 | 7632.120 | 82.082 | .000 | .472 | 140.931 | 1.000 |
| Lymph | Time * Group1 | Greenhouse-Geisser | 15.020 | 1.717 | 8.748 | .094 | .883 | .001 | .162 | .063 |
| Lymph | Error(Time) | Greenhouse-Geisser | 14687.261 | 157.959 | 92.981 |  |  |  |  |  |
| Neut | Time | Sphericity Assumed | 29079.408 | 2 | 14539.704 | 160.137 | .000 | .633 | 320.273 | 1.000 |
| Neut | Time * Group1 | Sphericity Assumed | 78.323 | 2 | 39.162 | .431 | .650 | .005 | .863 | .119 |
| Neut | Error(Time) | Sphericity Assumed | 16887.995 | 186 | 90.796 |  |  |  |  |  |
